# Diversity in *Chlamydial* plasmids

**DOI:** 10.1371/journal.pone.0233298

**Published:** 2020-05-29

**Authors:** Kolos V. Szabo, Colette E. O’Neill, Ian N. Clarke

**Affiliations:** 1 Faculty of Medicine, University of Southampton, Southampton, Hampshire, United Kingdom; 2 Molecular Microbiology Group, Clinical and Experimental Sciences, University Hospital Southampton, Southampton, Hampshire, United Kingdom; University of the Pacific, UNITED STATES

## Abstract

**Background:**

Evolutionary studies have been conducted that have investigated the chromosomal variance in the genus of *Chlamydia*. However, no all-encompassing genus-wide comparison has been performed on the plasmid. Therefore, there is a gap in the current knowledge on *Chlamydia* plasmid diversity.

**Aims:**

This project is aimed to investigate and establish the nature and extent of diversity across the entire genus of *Chlamydia*, by comparing the sequences of all currently available plasmid carrying strains.

**Methods:**

The PUBMED database was used to identify plasmid sequences from all available strains that met the set quality criteria for their inclusion in the study. Alignments were performed on the 51 strains that fulfilled the criteria using MEGA X software. Following that Maximum Likelihood estimation was used to construct 11 phylogenetic trees of the whole plasmid sequence, the individual 8 coding sequences, the iteron and a chromosomal gene *ompA* as a comparator.

**Results:**

The genus-wide plasmid phylogeny produced three distinct lineages labelled as alpha, beta and gamma. Nineteen genotypes were found in the initial whole plasmid analysis. Their distribution was allocated as six *C*. *pecorum*, two *C*. *pneumoniae*, one *C*. *gallinacea*, *one C*. *avium*, *one C*. *caviae*, *one C*. *felis*, two *C*. *psittaci*, *one C*. *trachomatis*, *one C*. *muridarum*, *and two C*. *suis*. The chromosomal comparative gene *ompA* supported this distribution, with the same number of primary clades with the same species distribution. However, *ompA* sequence comparison resulted in fewer genotypes due to a reduced amount of available sequences (33 out of 51). All results were statistically significant.

**Conclusion:**

The results of this study indicate that the common bacterial ancestor of all the species had a plasmid, which has diverged over time. Moreover, it suggests that there is a strong evolutionary selection towards these species retaining their plasmids due to its high level of conservation across the genus, with the notable exception of *C*. *pneumoniae*. Furthermore, the evolutionary analysis showed that the plasmid and the chromosome have co-evolved.

## Introduction

The *Chlamydia* are a distinct genus of pathogenic bacteria that can cause infections in humans and animals. Within this genus there are currently eleven recognised species [1] namely: *Chlamydia abortus*, *C*. *avium*, *C*. *caviae*, *C*. *felis*, *C*. *gallinacea*, *C*. *muridarum*, *C*. *pecorum*, *C*. *pneumoniae*, *C*. *psittaci*, *C*. *suis*, *and C*. *trachomatis*. *C*. *trachomatis* and *C*. *pneumoniae* are the most common chlamydial infections in humans, but zoonotic infections of *C*. *psittaci* and *C*. *abortus* [1] also occur. *C*. *abortus* is endemic among ruminants such as cows and sheep, but can also cause abortion in humans and other mammals. *C*. *pneumoniae* is a major cause of community-acquired pneumonia in humans, while *C*. *psittaci* is the cause of parrot fever (psittacosis), which may present as atypical pneumoniae that can mimic typhoid fever, but is often asymptomatic [2]. *C*. *trachomatis* is the most common bacterial sexually transmitted infection (STI) in humans [3], and it is the leading cause of preventable blindness in developing countries (trachoma) [4]. Less common syndromes and diseases caused by *C*. *trachomatis* include lymphogranuloma venereum [5] and reactive arthritis [6]. The remaining species have not been shown to cause disease in humans, but they can infect birds (*C*. *avium and C*. *gallinacea)*, cats (*C*. *felis*), koalas (*C*. *pecorum)*, rodents (*C*. *muridarum)* and swine (*C*. *suis and C*. *pecorum*).

All *Chlamydia* have a chromosome of around 1 Mbp. This is supplemented by a roughly 7.5 kbp circular plasmid in all species apart from *C*. *abortus* [7]. In the majority of microbial organisms, plasmids have a role in genetic variation and encode environment specific information. This can include antibiotic resistance which can be horizontally transferred and distributed among strains. Currently, there is no evidence of antibiotic resistance located on chlamydial plasmids. Plasmids are present in nine non-trachomatis species of *Chlamydia* (*C. avium [8], C. caviae [9], C. felis [10], C. gallinacea [8], C. muridarum [11], C. pecorum [12], C. pneumoniae [13–15], C. psittaci [16, 17], and C. suis [18–21]* and the human pathogen *C*. *trachomatis* [1]. Most chlamydial plasmids are 7.5 kbp in length with some exceptions [15, 22, 23]. They contain non-coding RNA with unknown functionality and 8 coding sequences (CDS) [24].

The origin of replication in chlamydial plasmids is thought to be regulated by iterons. Iterons are short repeated DNA sequences occurring between 3–5 iterations that play a crucial role in promoting replication of the plasmid [25]. Most *Chlamydia* species have four tandem repeat sequences, with each iteration comprising 22 base pairs [23, 26]. In *Chlamydia*, these repeat sequences have been described as mainly AT rich and have shown to be the most conserved regions by phylogenetic studies (reference?). This supports the notion that they play an important and conserved role in the regulation of plasmid replication.

Plasmid diversity within the genus *Chlamydia* was first investigated in twelve *C*. *psittaci* strains and with the prototype *C*. *trachomatis* L2 strain. Homology was recognised among the strains of *C*. *psittaci* [23]. However, he plasmid of *C*. *trachomatis* was the first characterised and consequently the most studied plasmid from the genus *Chlamydia*. Further studies established that these plasmids are present in most strains of *C*. *trachomatis*. The plasmid is highly conserved, with an intraspecific variation of around 3% [27]. Large-scale deletions or rearrangements of the plasmid are therefore rare [27, 28], but in 2006 a new strain was described that evaded PCR detection due to a single large deletion in CDS1 of its plasmid [29]. In 2018 157 whole genome sequences of *C*. *trachomatis* (including plasmid) isolates were deposited into the pubMLST database, where 902 genes were identified.[30] This was followed by the development of the MLST plasmid scheme that permitted the investigation of allelic variance. The results identified 6 plasmid clusters along with 4 chromosomal clusters. A close relationship was noted between the chromosomal genome, plasmid type and the diseases that they caused. They concluded that this would point towards co-evolution of the chromosome of *C*. *trachomatis* and their plasmids [31, 32].

Comparison of *C*. *psittaci* plasmid pCpA1 (avian), *C*. *pneumoniae* pCpnE1 (N16, equine) and *C*. *trachomatis* pMoPn (the agent of mouse pneumonia–this has been re-categorised into *C*. *muridarum*) and pLGV440 (human) further expanded the field [26]. This study showed maintenance of plasmid size at 7.5 kbp, although there was a single 200 nucleotide deletion in CDS1 of pCpnE1, which split it into two almost equal halves labelled ORF1A and ORF1B (CDS1A and CDS1B). This 200-nucleotide deletion proved to be the largest point of divergence from the other 3 strains. Furthermore, the analysis established the conservation of start codons for the 8 CDS’s between the plasmids: ATG (Methionine) for 1, 3, 4, 5 and 6, and GTG (Valine) for CDS 7 and 8. However, there is still debate over the actual start sequences of CDS 7 and 8 [26]. This highlights the ambiguity in the variance among these plasmids. The phylogenetic analysis concluded that MoPn was more closely related to *C*. *trachomatis* L1 (80%) than either the avian or equine strains.

The most extensive (non-*trachomatis*) phylogenetic study has been performed on *C*. *pecorum* plasmids [26]. In total 21 strains were used to characterise and compare the structure of the plasmids. The results showed a highly conserved plasmid with a low G+C content (31.6%). They varied in either 7,547 or 7,548 bp in length. The alignment of the 21 strains resulted in 12 unique plasmid genotypes labelled alphabetically from A to L. The 11 koala strains were identified into five genotypes, with SAK09Ure, SAK84Ure, and VicR6UGT Genotype A; NoHerEyes, PMHaUre, TedHUre and DbDeUG Genotype B; HazBoEye and HazBoUgt Genotype C; finally, Marsbar and IpTale Genotypes D and E, respectively. The three porcine strains were all Genotype L (R106, L1 and 1886). The four ovine strains were allocated into 3 plasmid genotypes, with p*Cpec*s CurE11Rec and CurE19Rec labelled as Genotype F, while remaining strains (W73 and IPA) were allocated into Genotypes G and H. The three bovine strains (WAB31Ileal, 66P130, and LW623) were of a unique genotypes, labelled I, J and K, respectively [12, 26].

Most recently, an epidemiological study of *C*. *gallinacea* compared a recently isolated *C*. *gallinacea* strain Jx-1 with type strain 08-1274/3 [33]. Jx-1 was found to contain a plasmid which was sequenced and deposited into GenBank. pJx-1 shared 99.9% identity to p1274 with only two point mutations at position 6573 (C to T) and 7170 (C to A) in PGP 6 reflecting the close relatedness of these two strains.

Phylogenic studies have been performed both within and between species, however, at the present time no all-encompassing genus-wide comparison has been performed. Advances in sequencing technology means there is now an abundance of plasmid sequences available for all known species in public databases. It is time to draw all this information together. Therefore, the aim of this study was to investigate the nature and extent of diversity across the non-trachomatis chlamydial species, by analysing all currently published plasmid-carrying strains.

## Materials and methods

### Plasmid sequence data

Whole genome sequences of non-trachomatis species that were available in PubMed’s Nucleotide database were included for analysis. Furthermore, due to the large deletion in the only available peer-reviewed WGS of *C*. *suis* strain MD56, 3 directly submitted (unpublished) *C*. *suis* strains were used to provide greater insight into the significance of the deletions present on MD56. In total 50 non-trachomatis *Chlamydia* strains have been identified to have a plasmid (Table 1). The *C*. *trachomatis* LGV440 (L2) was included for comparative reasons, as it has been widely studied.

**Table 1:** List of all the Chlamydial species that are being used for the study. Relevant information on length of plasmid, accession number, PUBMED ID and source provided.

Genus and Strain	Length in BP	PUBMED	Acession
***C*. *avium***			
10DC88	7099	24461712	CP006572.1
***C*. *caviae***			
GPIC (pCpGP1)	7966	12682364	AE015926.1
***C*. *felis***			
C-56 (pCFe1)	7552	16766509	AP006862.1
***C*. *gallinacae***			
Jx-1	7492	29212448	CP019793.1
08-1274/3	7619	24461712	CP015841.1
***C*. *muridarum***			
Nigg (pMoPn)	7501	10684935	AE002162.1
***C*. *pecorum***			
66P130	7548	26870613	KT223766
1886	7548	26870613	KT223767
CurE11Rec	7547	26870613	KT223768
CurE19Rec	7547	26870613	KT223769
DbDeUG	7547	26870613	KT223770
IPA	7547	26870613	KT223771
IpTaLe	7547	26870613	KT223772
LW623	7547	26870613	KT223774
SaK09Ure	7547	26870613	KT223777
SaK84Ure	7547	26870613	KT223778
VicR6UGT	7547	26870613	KT223779
L1	7548	26870613	KT223773
R106	7548	26870613	KT223776
Marsbar	7547	26870613	KT223775
W73	7547	26870613	KT223780
HazBoEye	7547	26870613	KT352920
HazBoUGT	7547	26870613	KT352921
NoHeEye	7547	26870613	KT352922
PMHaUre	7547	26870613	KT352923
TedHUre	7547	26870613	KT352924
WaB31Ileal	7548	26870613	KT223781
***C*. *pneumoniae***			
N16 (pCpnE1)	7368	9202459	X82078.1
B21	7533	24503994	AZNB01000165.1
LPCoLN	7530	19749045	CP001714.1
***C*. *psittaci***			
84/55	7487	23209198	CP003812
CB7	7577	24903864	JMBZ01000059.1
HJ	5258	25744990	JPIH02000081.1
6BC	7553	21441521	CP002550.1
Cal10	7553	21622741	AEZD01000004.1
CP3	7552	23209198	AFVN01000005.1
M56	7553	23209198	CP003814.1
NJ1	7552	23209198	AFVK01000004.1
WC	7553	23209198	CP003818.1
VS225	7553	23209198	CP003817.1
MN	7491	23209198	CP003815.1
WSRT30	7553	23209198	CP003819.1
DD34	7553	25887617	AFVL01000005.1
Frances	7553	25887617	AFVM01000003.1
UGA	7553	25887617	AWXQ01000007.1
RD1	7553	21183672	FQ482150.1
***C*. *suis***			
MD56	5976	24812227	AYKJ01000001.1
SWA-2	7494	Direct submission	LT821324.1
SWA-14	7494	Direct submission	LT860208.1
SWA-86	7494	Direct submission	LT860208.1
***C*. *trachomatis***			
LGV440	7500	2836808	X06707.3

For each plasmid, their corresponding chromosomal major outer membrane protein (ompA) gene was selected for comparative phylogenetic analysis. This gene was chosen as it has been extensively studied with established variance present across interspecies analyses [31]. Thirty-three of the 51 plasmids sequences were identified with a corresponding *ompA* gene in the database.

### Phylogenetic analysis

MEGA X (version 10.0.5) was used to execute alignments. Alignment were performed with ClustalW, following which a Maximum Likelihood tree was constructed for each CDS. The Maximum Likelihood model selects trees and branches based on the highest probability of them forming. The overall probability for each tree is presented in a log-likelihood function. General likelihood values are small, therefore applying them in log function will present a more distinguishable data set. However, these results will be negative due to the values being less than one in the function. This method assists in the reconstruction of the evolutionary history of these plasmids and might reveal possible recombination. The eight coding sequences (CDS) were individually compared, which made it possible to identify individual gene recombination events as branch swaps on the constructed phylogenies. The 51 whole plasmid sequences were then aligned and a ML phylogenetic tree was constructed. Finally, the same phylogenetic method was used to establish the diversity within the iteron across the extracted plasmids.

SnapGene (version 4.2.7) was used to extract each CDS from whole plasmid sequence data. An assumption was made that most of these genes would be labelled uniformly. The following species had the correct labelling *C*. *caviae*, *C*. *felis*, *C*. *muridarum*, and *C*. *pneumoniae*. The rest had errors ranging from incorrectly labelling CDS1 as CDS2 (all the *C*. *pecorum* strains), to missing labels which required the use of a diagram based on *C*. *pneumoniae* LPCoLN to establish the correct CDS (Table 1).

The phylogeny of all the alignments was deduced using the Maximum Likelihood method with the Tamura-Nei nucleotide substitution model with uniform rates. The phylogeny test was statistically supported by 1000 bootstrap replications to provide the probability of each branch's formation. Bootstrapping is a method of re-sampling each branch to provide a confidence to each outcome [34]. Essentially it illustrates the probability of each branch being recovered if the taxa were sampled multiple times. As recommended [34], the parameters were set for 1000 repetitions and the cut off value of x ≥70% was used, due to studies [35] showing that it corresponds to a 95% confidence that the clade is real. Thus, any value above 70% (0.7) would suggest a statistically significant result. For tree inference options the initial tree was automatically formed, and nearest-neighbour-interchange was applied for the heuristic method. No outgroup was selected for the analysis of the divergence.

Mega X was further used to perform the p-distance analysis on nucleotide level, with 1000 bootstrap repetitions to determine the standard error (SE). Gaps were considered as complete deletions. The model to determine substitutions was set as the p-distance method, with substitutions type selected as nucleotide and data to include transitions, transversions, and deletions.

## Results

### Structural composition of plasmids

The alignments of the plasmid sequences provided the initial results on the organisational differences between strains. Structurally, most strains adhere to the currently accepted model for chlamydial plasmids (Fig 2). With a few exceptions, they all contain eight coding sequences (CDS) that can be found at very similar spatial locations. The direction of transcription for each gene is conserved with CDS2 being the only gene that is inversely transcribed.

This conforms to the idea that these 8 CDSs play a significant role in the function of the bacteria, due to their preservation across all species. However, the current study indicates significant variation in the following strains: *C*. *avium 10DC88*, *C*. *gallinacea* 08-1274/3, *C*. *pneumoniae N16*, *C*. *psittaci HJ*, *and C*. *suis MD56* (Fig 2). Complete gene deletion occurs in *C*. *avium* at CDS5 and CDS6.

It has been hypothesised that the plasmid has a role in virulence, with genes CDS5 and CDS6 being the most likely candidates, making them essential for infection and propagation *in vivo* [36] However, both CDS5 and CDS6 have been proven to be redundant in the survival of a plasmid, illustrated by the ability of *C*. *avium* 10DC88 to lose both of these genes. Furthermore, both CDS5 and CDS6 are dispensable for *Chlamydia* propagation in *in vitro* studies [37]. Moreover, *C*. *avium 10DC88* has an early stop codon that partitions CDS2 into two separate genes, identified as 2A and 2B. Due to the absence of more examples of *C*. *avium* plasmid sequences, it is not possible to (a) check the validity of the data and (b) determine the origin of this mutation that gives rise to this early stop codon.

**Fig 1:**
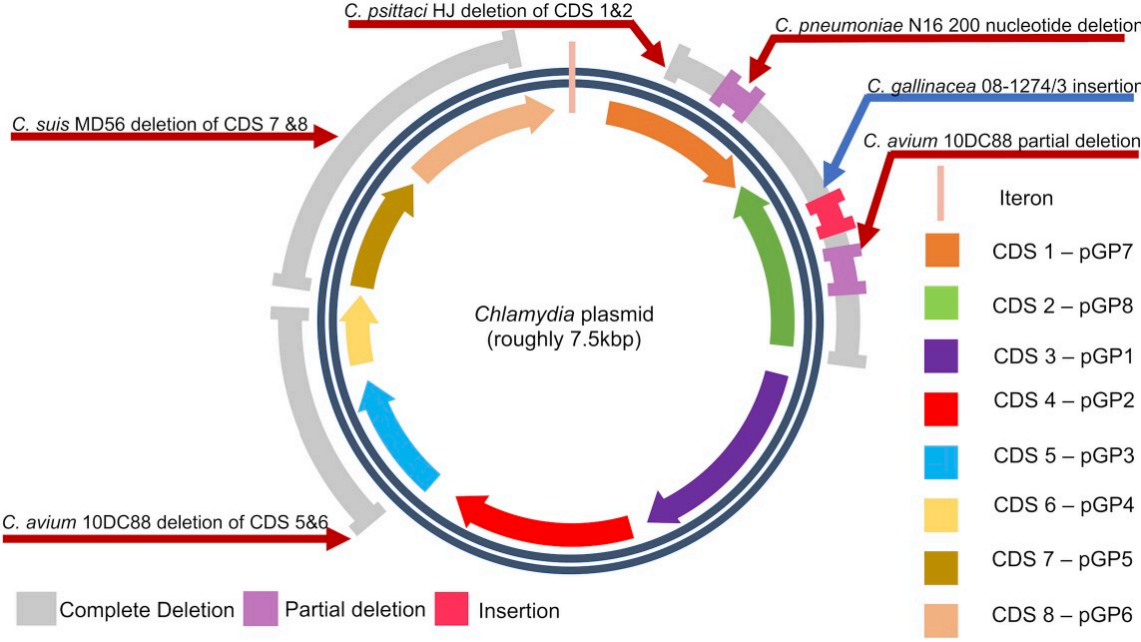
General structure of chlamydial plasmids with annotated mutations and their strain names.

However, the whole plasmid phylogeny (Fig 1) provides us with the tool to investigate the nature of this deletion by examining the relatedness of *C*. *avium’s* sequences to those of *C*. *gallinacea*. When compared to *C*. *gallinacea* JX-1, there is a single nucleotide deletion that caused a frameshift and the codon for lysine is replaced with a stop codon. *C*. *gallinacea* 08-1274/03 also has an early stop codon in CDS 2, caused by a 62-nucleotide insertion into the middle of the gene. As in *C*. *avium*, the consequence of this is the formation of two separate genes labelled 2A and 2B. *C*. *psittaci HJ* has the largest deletion among all strains, resulting in the loss of CDS 1 and CDS 2, which makes it the smallest plasmid. Furthermore, *C*. *suis* MD56 has a complete deletion of CDS 7 and CDS 8, a feature which is absent in the directly submitted *C*. *suis* samples. This would suggest that this is not a fixed mutation in *C*. *suis*, and is instead unique to MD56. CDS 7 translates into a protein that is involved in the partitioning of the plasmid, whilst CDS 8 has a function in the control of replication. The apparent importance of these genes to plasmid maintenance and the absence of these mutations in the directly submitted sequences may lead us to question the authenticity of the *C*. *suis* MD56 sequence. However, in the event that the deletions in MD56 are genuine, they would indicate that those genes are redundant and that there may be failsafe mechanisms to substitute for those mutations in the plasmid. Finally, *C*. *pneumoniae* plasmid pCpnE1 contains a 200-nucleotide deletion in CDS 1 that has resulted in the production of two distinct CDSs labelled 1A and 1B. This was previously identified in 1997 [15], and the validity of this mutation is corroborated by a similar deletion in the Swedish New Variant *C*. *trachomatis* strain, supporting the notion that CDS2 compensates for loss of CDS1 activity [29].

### Whole plasmid phylogeny

The results for each phylogenetic tree are listed with their highest log likelihood, average p-distance with SE, and number of alleles constructed (Table 2). Each tree has been condensed to show only values of and above 70 (0.7). Therefore, all the clades that have been generated are statistically significant [35].

**Table 2:** Annotation of each element of the plasmid. This includes the putative function and length of each element. The table includes the results of the average p-distance, alleles present and log likelihood of tree formation.

Annotation	Hypothetical function	Base pair length range	Mean P-distance	SE	Alleles	Phylogeny log(x)
CDS1/pgp7	Integrase	252–1017	0.248	0.007	14	-7012.49
CDS2/pgp8	Integrase	450–1068	0.254	0.008	12	-5960.31
CDS3/pgp1	Replicative DNA helicase	1298–1422	0.26	0.006	14	-10908.87
CDS4/pgp2	Virulence plasmid protein	924–1065	0.172	0.007	14	-6799.12
CDS5/pgp3	Virulence plasmid protein	795–825	0.278	0.008	14	-6281.03
CDS6/pgp4	Virulence plasmid protein	306–209	0.181	0.012	8	-1728.8
CDS7/pgp5	Plasmid partitioning protein	627–810	0.254	0.01	15	-5880.2
CDS8/pgp6	Plasmid replication protein	252–1017	0.274	0.009	14	-6252.29
Repeat sequence	Iteron	88bp(22x4 tandem repeat)	0.017	0.005	4*(1 incomplete)	-215.66
Whole Plasmid	-	5258–7966	0.5	0.001	19	-60175.44
Chromosomal ompA	Membrane transporter	1062–1209	0.24	0.008	15	-11947.12

**Fig 2:**
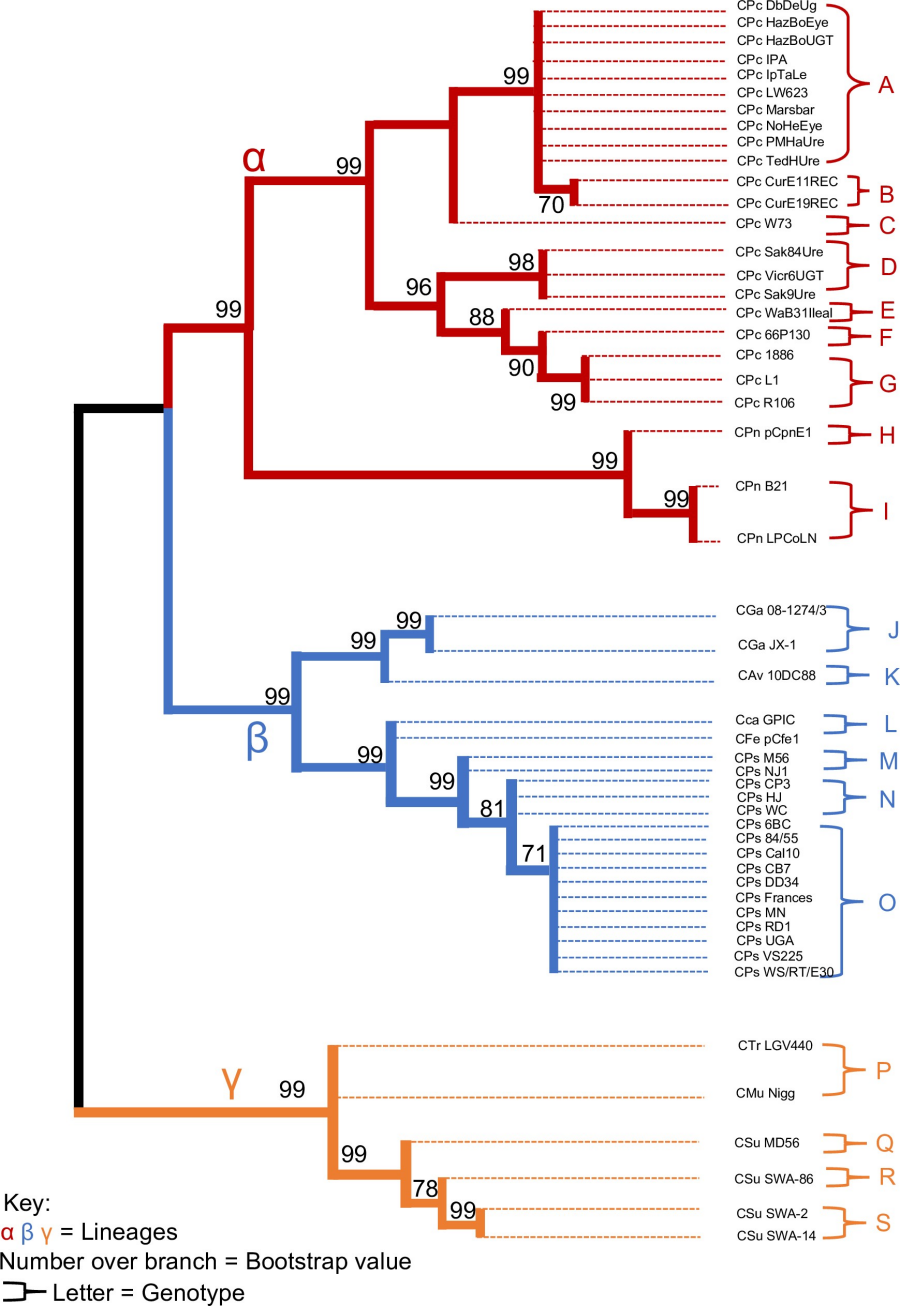
Maximum Likelihood estimate phylogeny for 51 Chlamydial plasmids with a log likelihood of log(-60175.44).

The entire plasmid phylogeny produced three distinct lineages that are labelled as alpha, beta and gamma (Fig 2). For the total number of 51 plasmids, the corresponding 19 genotypes were found in the initial whole plasmid analysis (Table 2). Their distribution was allocated as follows: seven *C*. *pecorum* (A, B, C, D, E, F and G), two *C*. *pneumoniae* (H and I), one *C*. *gallinacea* (J), one *C*. *avium* (K), one *C*. *Caviae* (L), one *C*. *felis* (M), two *C*. *psittaci* (N and O), one *C*. *trachomatis* (P), one *C*. *muridarum* (Q), *and* two *C*. *suis* (R and S). The chosen chromosomal comparator gene *ompA* had the same number of primary clades. However, it resulted in fewer genotypes, the total number being 15 for the 33 sequences that were available.

In terms of intraspecies analysis, the 21 individual *C*. *pecorum* strains were allocated into 7 distinct genotypes. *C*. *pecorum* porcine plasmids 1886, L1, and R106 were identified in the same clade and were annotated as genotype G with a 0.99 bootstrap value. The largest plasmid group, labelled genotype A, contained the following 10 strains; DbDeUG, HazBoEye, HazBoUGT, IPA, IpTaLe, LW623, Marsbar, NoHeEye, PMHaUre, and TedHUre. The remaining three Koala strains Sak09Ure, Sak84Ure, and Vicr6UGT were identified in the same genotype labelled D. A subclade was formed for the two ovine strains CurE11REC and CurE19REC, identified as genotype B. The remaining 3 strains (W73, WaB31Ileal, and 66P130) were identified as distinct plasmid types (C, E and F respectively).

**Fig 3:**
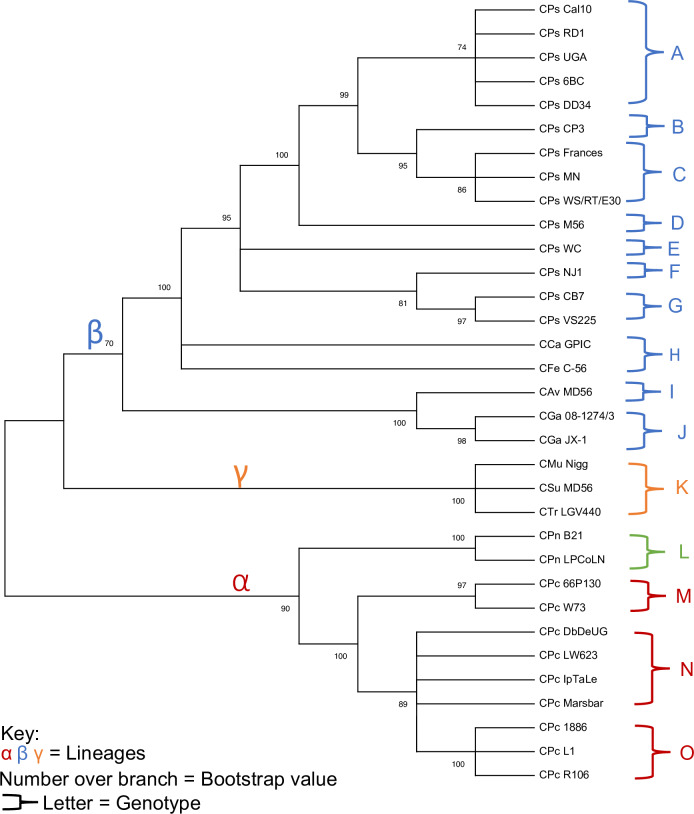
The Maximum Likelihood estimate for 33 ompA strains with a log likelihood of log(-11947.22).

The *ompA* phylogeny (Fig 3) resulted in the equal number of primary clusters (labelled alpha, beta, and gamma), which were all allocated with the same species as the whole plasmid phylogeny.

### Coding sequence phylogenies

**Fig 4:**
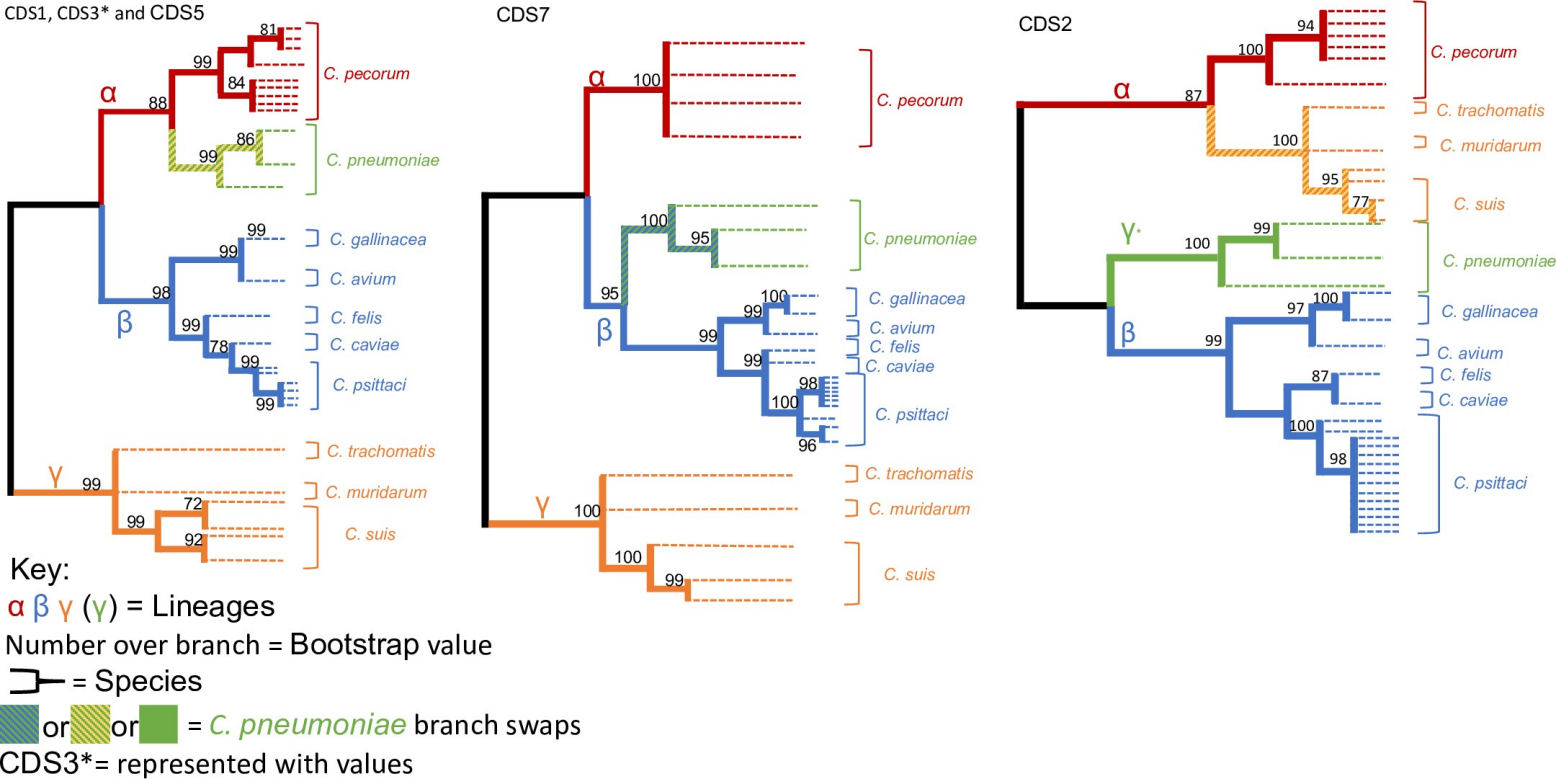
The Maximum Likelihood estimate for gene group A with the following five coding sequences being present; CDS1, CDS2, CDS3, CDS5, and CDS7.

All 8 coding sequences were further analysed to obtain a higher resolution of the extent of divergence. The ML tree for the 8 genes was subcategorised into two groups (A&B) according to their number of lineages. Group A (Fig 4) accommodated CDS1, CDS2, CDS3, CDS5, and CDS7 (pgp 7, 8, 1, 3, and 5). All 5 genes supported 3 lineages, with CDS1, CDS3, and CDS5 mirroring the distribution of species seen in the whole plasmid phylogeny. The remaining two genes each had a major differentiating point that distinguished themselves as unique from the rest of the group and one another. CDS2 had the largest point of divergence with *C*. *pneumoniae* forming the third gamma lineage by itself, supported by a 1.00 bootstrap value. This led to the migration of the cluster that contains *C*. *muridarum*, *C*. *suis*, *and C*. *trachomatis* to the alpha lineage (0.87 bootstrap value), hence suggesting a closer relationship with *C*. *pecorum* species than the others. CDS7 had a less dramatic change to its structure with *C*. *pneumoniae* crossing over to the Beta cluster (1.00 bootstrap support), thus leaving *C*. *pecorum* on its own in the alpha cluster. Within group A, CDS2 had the lowest number of genotypes at 12. Conversely, CDS7 was identified to have the most genotypes with 15. The remaining three CDS’ (CDS1, CDS3 and CDS5) generated 14 unique genotypes.

**Fig 5:**
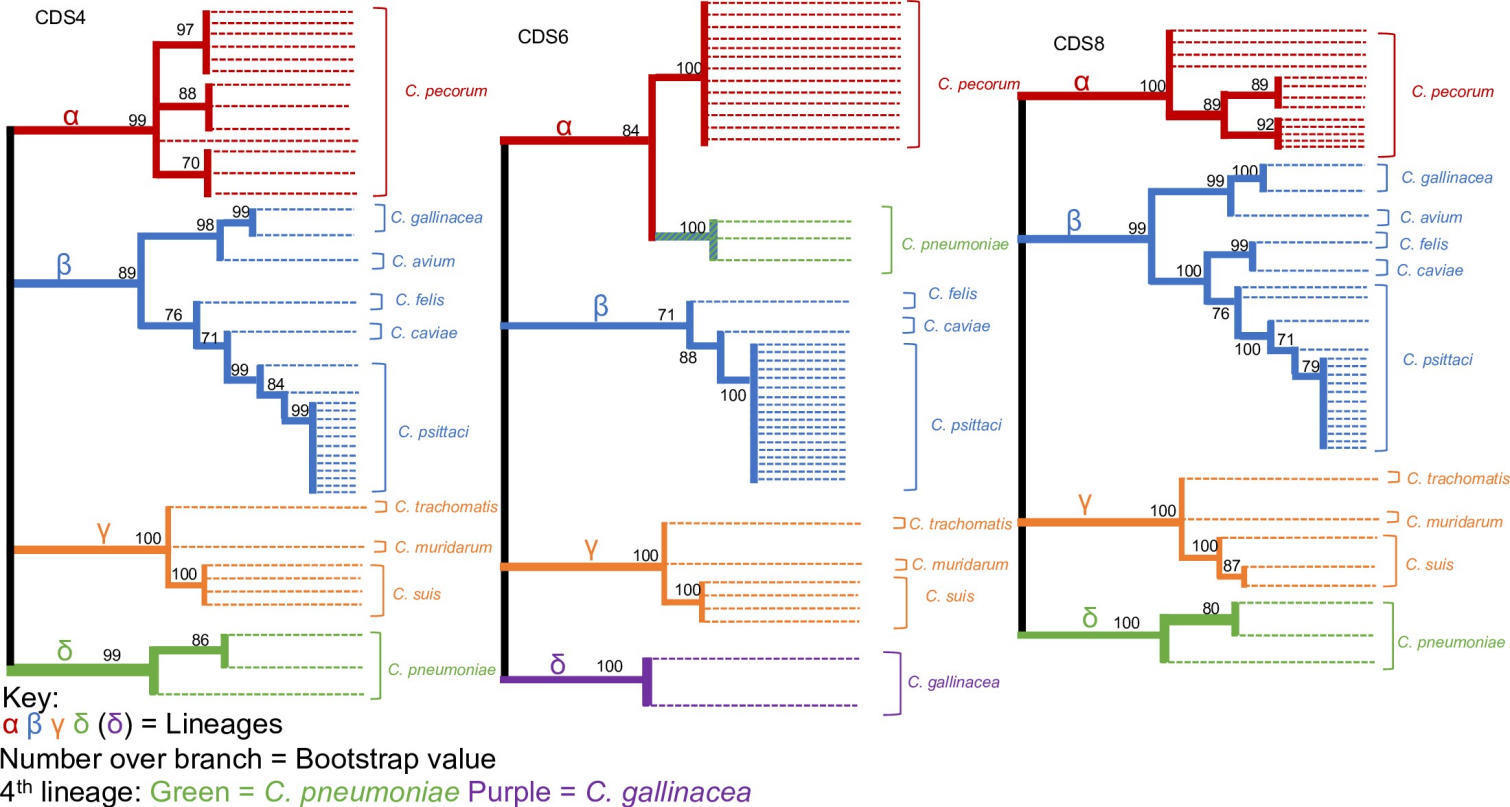
The Maximum Likelihood estimate for gene group B with the following 3 coding sequences being present; CDS4, CDS6, and CDS8.

CDS4, CDS6 and CDS8 were allocated into Group B (Fig 5), which is categorised by the emergence of a 4^th^ primary cluster, labelled delta. CDS 4 and 8 have *C*. *pneumoniae* as their 4^th^ delta lineage, with a bootstrap value of 0.99 and 1.00 respectively. Considering the previously discussed branch swap in CDS7, and the unique cluster formation in CDS2, it could be possible that *C*. *pneumoniae* is a common ancestor from which other species have diverged. However, it is also possible that recombination has occurred with some of its genes.

Both CDS 4 & 8 had 14 genotypes with each species having its own unique sequence. Analysis of CDS4 identified *C*. *gallinacea* as a 4^th^ lineage with a 1.00 bootstrap value. *C*. *pneumoniae* was located on the alpha cluster, conforming to the structure of the whole plasmid phylogeny. CDS6 was divided into 8 sequence types, which was the lowest across all CDSs.

### Iteron phylogeny

**Fig 6:**
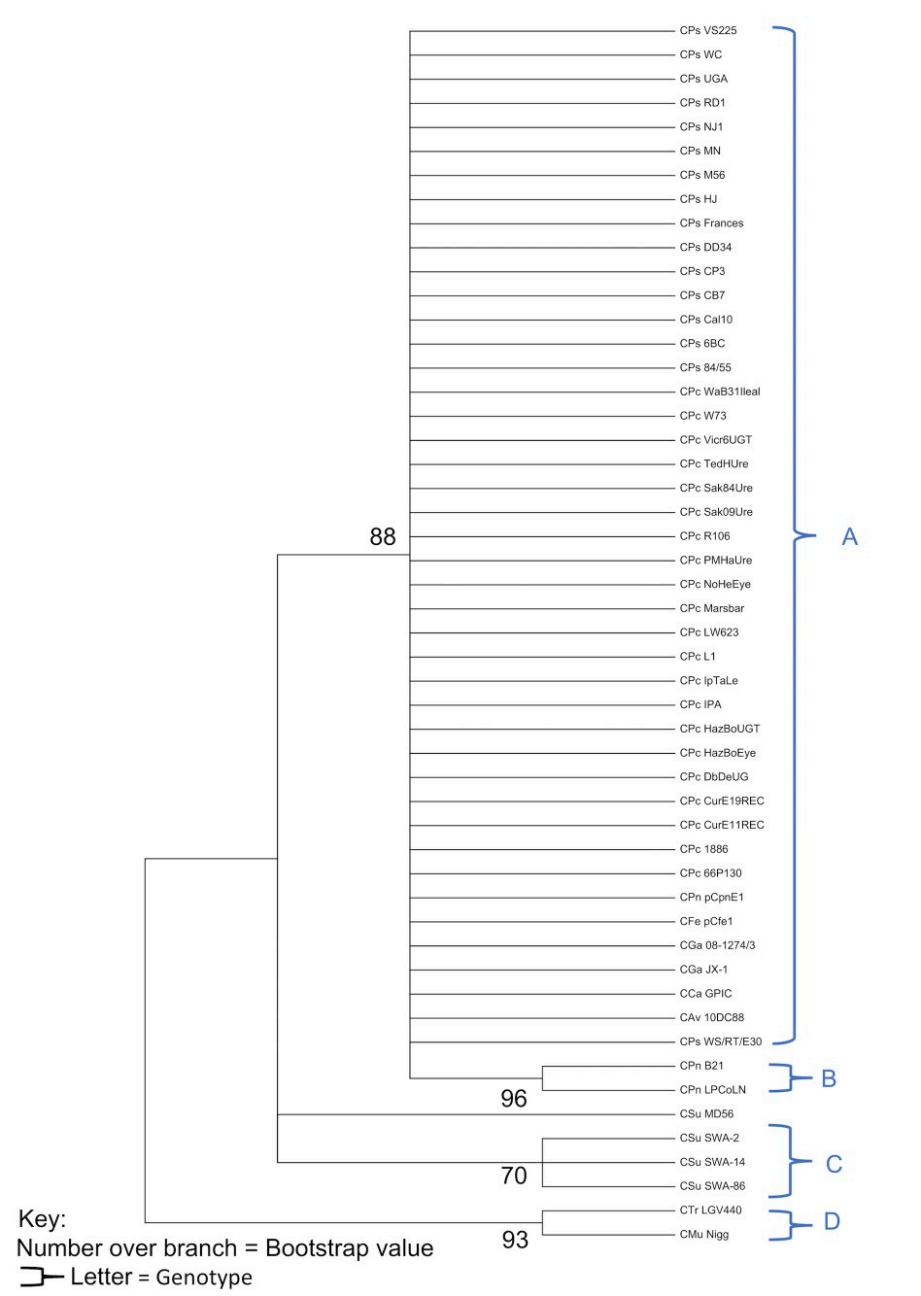
The maximum likelihood estimate for 51 iteron plasmid strains with a log likelihood of log (-215.66).

The iteron was shown to be the most highly conserved sequence of the plasmid across the species. 43 of the 51 plasmids were identified as identical with a 0.88 bootstrap value (Fig 6). These 43 strains consist of all the *C*. *pecorum*, *C*. *psittaci*, *C*. *gallinacea*, *C*. *avium*, *C*. *caviae*, *C*. *felis* plasmids and one *C*. *pneumoniae* strain pCpnE1 (N16) plasmid. The plasmids from the remaining *C*. *pneumoniae* strains formed a sub-clade with a 0.96 bootstrap value. *C*. *suis* formed its own clade with the strains SWA-2, SWA-14, and SWA-84. Due to a large deletion leaving it with a 2-tandem repeat, *C*. *suis* strain MD556 was identified within its own cluster. Finally, *C*. *trachomatis* and *C*. *muridarum* were shown to have a similar iteron. Despite the formation of 4 clades in the phylogenetic tree, the differences between these sequences are extremely minor as shown by the average p-distance of 0.017 (SE = 0.008) (Table 2), which is the lowest across all elements of the plasmid. The high degree of conservation of the iteron among chlamydial species strongly suggests its importance in the regulation of replication of *Chlamydial* plasmids.

### Pairwise matrices

Pairwise matrix computation was performed on each CDS to determine the most and least conserved genes (Table 2). The average p-distance, with standard error (SE) in brackets (x), were the following (starting with CDS1 and ending with CDS8 in numerical order: 0.248 (0.007), 0.254 (0.008), 0.260 (0.006), 0.172 (0.007), 0.278 (0.008), 0.181 (0.012), 0.254 (0.01), and 0.274 (0.009). These results identified CDS4 as the most conserved and CDS5 as the least conserved gene across the sampled species. Due to mutations in CDS2, the test was recalculated with the elimination of the mutated strains to test the difference they make across the species. The results showed a very minor difference, with a p-distance of 0.246 and an unchanged SE.

## Discussion

Advances in genome sequencing technology have provided significantly more data to enhance our understanding of the evolution and phylogeny of the chlamydial plasmid. The availability of open databases has provided us with seamless access to all currently sequenced strains.

The structural distribution of the genes in the plasmid, and their direction of transcription was conserved. This is a clear indicator that the plasmid is essential for the survival of the organism in plasmid-bearing species. However, the presence of mutations indicates that some parts of the plasmid are potentially redundant. The pairwise distance matrices analysis provides necessary information about which CDSs and genetic elements are the most conserved. This identified CDS4 as the most conserved (p-distance of 0.181 with SE = 0.012) and CDS5 (p-distance of 0.278 with SE = 0.008) as the least conserved gene across the genus (Table 2). This analysis conforms with the previously studied hypothesis that identified CDS5 (virulence associated protein) as the most polymorphic gene within *C*. *trachomatis* with a p-distance of 0.008 [30]. However, the same study identified CDS2 as the most conserved and least diverse gene on the plasmid with only 11 allelic variations within the *C*. *trachomatis* species [30], whilst a more recent study identified CDS6 as the most conserved plasmid gene in *C*. *trachomatis* across a much larger sample set (but CDS2 was the most conserved at the amino acid level) [27] Our calculations gave a p-distance of 0.254 and SE = 0.008 for CDS2, which was considerably higher compared to CDS4 with a p-distance of 0.181. One might argue that this difference could be due to two of the present species (*C*. *avium* 10DC88 and *C*. *gallinacea* 08-1274/3) possessing mutations. However, with their elimination, the mean distance only decreased to 0.246. The virulence associated CDS4 is the most conserved plasmid gene on a genus-wide spectrum. This means that CDS4 has an integral role in chlamydial survival and/or pathogenesis as nature selects the retention of this gene with little diversity.

### Lineages

The phylogenetic tree for the whole plasmid sequences clearly illustrates three distinct and well-supported lineages, labelled as alpha, beta, and gamma (Fig 1). The alpha lineage consists of *C*. *pecorum* and *C*. *pneumoniae*. Our study was able to categorise the 21 strains of *C*. *pecorum* into 8 unique plasmid genotypes (alphabetically from A to L). This is different from a previous study [26], which predicted 12 plasmid types. This could be attributed to the differences in phylogenetic techniques used, as Bayesian inference can produce different results compared to Maximum likelihood estimations [34].

The koala LPCoLN and bandicoot B21 *C*. *pneumoniae* strains were clustered in the alpha clade (I), while equine pCpnE1 (N16) strain formed its own genotype H. From an evolutionary point of view, the alpha lineage is strongly rooted in domesticated mammals, with strains infecting bovine, equine, ovine and porcine hosts. Koalas have been infected with both *C*. *pecorum* and *C*. *pneumoniae*, despite being undomesticated wild animals. This supports the hypothesis that the koala habitat overlaps with farmland and transmission between domesticated (farm) animals and koalas is occurring [38].

The beta lineage contains *C*. *avium*, *C*. *caviae*, *C*. *gallinacea*, *C felis*, *and C*. *psittaci*. Within this group, *C*. *avium* and *C*. *gallinacea* were most closely related with a 0.99 bootstrap value. However, they ended up forming separate plasmid types, seen as J for *C*. *gallinacea* and K for *C*. *avium*. Phylogeny pointed towards three clades (M, N and O) within *C*. *psittaci*, of which clade O had the following 11 strains: p*Cps* 6BC, 84/55, Cal10, CB7, DD34, Frances, MN, RD1, UGA, VS225, and WS/RT/E30. Clade M contained the pigeon strain HJ and muskrat strain M56. The remaining strains belonged to genotype N. Finally, both *C*. *caviae* and *C*. *felis* were shown to have a closer relationship with *C*. *psittaci* than the clade formed under *C*. *avium* and *C*. *gallinacea*, hence they were classified into clade L. This whole cluster mainly infects avian species such as poultry and psittacine birds. However, they also appear to be more related to cat and guinea pig specific *Chlamydia*. A deeper evolutionary history, in the form of more strains, is required to improve our understanding of how *C*. *caviae* and *C*. *felis* are related to avian strains.

The final lineage gamma was comprised of the following species; *C*. *muridarum*, *C*. *suis*, and *C*. *trachomatis*. *C*. *muridarum* and *C*. *trachomatis* were allocated into clade P. *C*. *suis* was shown to have 3 different plasmid types (Q, R and S). p*Csu* MD56 would obviously be presented as a unique plasmid (Q) due to the ≈2kbp deletion of CDS 7 and CDS 8. Of the remaining strains, SWA-2 and SWA-14 were predicted as identical (hence identified as plasmid S), and SWA-84 as a unique plasmid R. The relatedness of these three chlamydial species can be attributed to the habitat of their host species. As humans have progressed from their more tribal history, their contact with Muridae and Porcine species has increased. However, this study is limited in the ability to suggest the direction that evolution has taken. This can be improved by changing the technique used for the construction of the phylogenetic trees. Rather than using the bootstrap method to estimate the probabilistic significance of the results, average p-distance can be used with a rooted group. The rooted group would have to be an assumed or proven ancestor, which would provide information about the direction that natural selection has taken.

When compared to the *ompA* phylogeny (Fig 3) the maximum likelihood estimation resulted in a perfectly mirrored image of the whole plasmid, suggesting that the chromosome and plasmid have co-evolved. This is consistent with earlier studies that have investigated the diversity within the *C*. *trachomatis* species[32]. However, a caveat is that the smaller sample size of the *ompA* genes is a limiting factor in this analysis. Due to the presence of only 33 sequences out of the possible 51 (at the time of this study), it would be a challenge to determine how well the genotypes of *ompA* sequences compare to that of the plasmid. For future work, sequencing of the *ompA* of the 18 missing strains would help form a more accurate representation of the similarity between the chromosome and plasmid diversity.

Irrespective of lineage, all *Chlamydia* species carrying a plasmid have consistently had a unique plasmid genotype compared to other species. This further supports the notion that *Chlamydial* plasmids are highly conserved on the level of the entire plasmid and that they have co-evolved with the chromosome[32]. However, this type of preservation is not unanimously present when analysing single genes. The individual CDS ML estimations provided us with two distinct phylogeny types that were allocated as Group A (Fig 4) and Group B (Fig 5). These two phylogeny types were determined by the number of primary clusters they generated. Group A closely resembled the structure of the whole plasmid with 3 lineages, while Group B has an extra 4^th^ cluster. The main discussion point for both groups is the nature of *C*. *pneumoniae’s* branch swap, and whether it is a main ancestor and point of evolutionary direction. This study is aimed at identifying points of divergence, therefore rooting of another hypothesised common ancestor of *Chlamydia* would be required to answer this question. However, these results demonstrate that the *C*. *pneumoniae* plasmid is unique compared to the others as it can be located in different lineages dependant on the gene.

### Plasmid loss and acquisition

There is no evidence for acquisition of a specific plasmid from another chlamydial species based on the current animal chlamydial plasmids in the databases, this may be because of the relatively small sample size and that such events are rare and have yet to be uncovered; indeed, sequencing of a much larger sample of *C*. *trachomatis* genomes revealed occasional evidence for plasmid transfer between clades [39]. In the most well studied biological situation, we know that *C*. *trachomatis* plasmids cannot by themselves replicate in the closely related *C*. *muridarum*, the barrier is at the level of plasmid replication and the *C*. *trachomatis* plasmid must acquire the CDS2 from the *C*. *muridarum* plasmid to replicate in the *C*. *muridarum* host [40]. *C*. *abortus* is the only species that universally does not carry a plasmid. A phylogenetic tree rooted on *C*. *psittaci* showed the genomes of all the *C*. *abortus* isolates so far sequenced fall into two long branches [41]. This analysis also postulates that *C*. *abortus* evolved from a plasmid-bearing ancestor of *C*. *psittaci*. In this work, ‘dating’ the origins of the *C*.*abortus* species within the genomic phylogeny was based on estimated mutation rates, suggesting the origins of *C*. *abortus* can be traced back in millennia and thus could coincide/approximate to the domestication of sheep, some 9–10,000 years ago. The complete absence of plasmids and limited relative diversity in *C*. *abortus* genomes (in comparison to other chlamydial species) indicates that the transmission of the *C*. *abortus* ancestor to domesticated sheep probably happened once (or a few times), and was co-incident with plasmid loss. A genetic explanation for the continued pathogenicity of *C*. *abortus* in the face of plasmid-loss still eludes evolutionary studies.

A further point of interest is that plasmids have not been found in any human isolates of *C*. *pneumoniae* sequenced so far [42]. Human *C*. *pneumoniae* genomes are extremely homologous suggesting a more recent movement of the pathogen from animals to humans than the emergence of *C*. *abortus* from *C*. *psittaci*. It has been proposed that humans were infected by an animal isolate of *C*. *pneumoniae* which then adapted to humans by gene decay and plasmid loss [43]. It seems likely that this ‘jump’ to humans occurred possibly only once and was accompanied by the loss of the ancestral *C*. *pneumoniae* plasmid, then the strain spread through the human population. Recently we developed a vector-based genetic transformation system for *C*. *pneumoniae* using the plasmid that came from the equine isolate of *C*. *pneumoniae* N16 [44]. This recombinant equine plasmid can replicate efficiently in human *C*. *pneumoniae* isolates and whilst human *C*. *pneumoniae* have undergone gene decay, they have clearly not lost the ability to support the replication of an animal *C*. *pneumoniae* plasmid. This equine plasmid presumably originates from a *C*. *pneumoniae* that was a common ancestor for current human and animal *C*. *pneumoniae*. Intriguingly it was also shown that the recombinant equine *C*. *pneumoniae* plasmid could replicate in several strains of *C*. *felis* [44]. Whilst the chromosomes and plasmids of chlamydial species have co-evolved to the extent that the plasmids are species-specific/exclusive *C*. *felis* has yet to reach that level of divergence and so can support the replication of plasmids from other Chlamydia without the need for acquisition of accessory plasmid sequences from the recipient host plasmid.

## Conclusions

This phylogenetic study provides a framework for investigating the biological properties of the chlamydial plasmid. The phylogeny divided the genus into three distinct lineages designated alpha, beta and gamma. Our results indicate that the lineages diverged from a common ancestor of all the chlamydial species that carried a plasmid. Moreover, there is evidently a strong evolutionary selection towards these species retaining their plasmids since almost all species within the Chlamydiaceae carry a plasmid. No evidence was found for interspecies plasmid transfer.

## Supporting information

S1 Fig Maximum-Likelihood estimate for CDS1. Raw unannotated MEGA X data.
(TIF)

S2 Fig Maximum-Likelihood estimate for CDS2. Raw unannotated MEGA X data.
(TIF)

S3 Fig Maximum-Likelihood estimate for CDS3. Raw unannotated MEGA X data.
(TIF)

S4 Fig Maximum-Likelihood estimate for CDS4. Raw unannotated MEGA X data.
(TIF)

S5 Fig Maximum-Likelihood estimate for CDS5. Raw unannotated MEGA X data.
(TIF)

S6 Fig Maximum-Likelihood estimate for CDS6. Raw unannotated MEGA X data.
(TIF)

S7 Fig Maximum-Likelihood estimate for CDS7. Raw unannotated MEGA X data.
(TIF)

S8 Fig Maximum-Likelihood estimate for CDS8. Raw unannotated MEGA X data.
(TIF)
