# Supplementary figures and images for: Diversity in Chlamydial plasmids
Source: PLoS One. 2020 May 29;15(5):e0233298. doi: 10.1371/journal.pone.0233298 (PMC7259575; doi:10.1371/journal.pone.0233298)

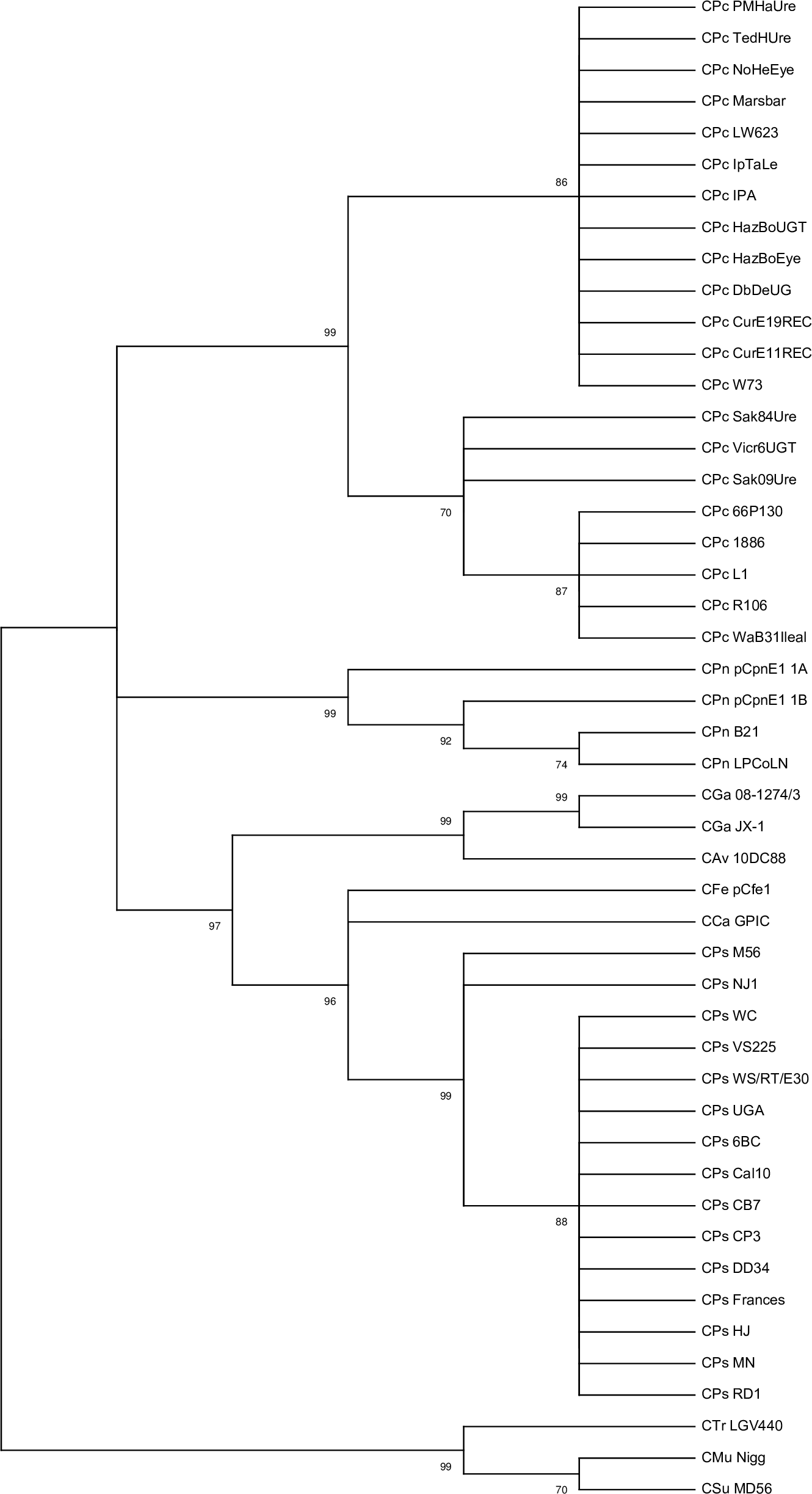

Supplement: S1 Fig — Raw unannotated MEGA X data. (TIF) [file pone.0233298.s001.tif]

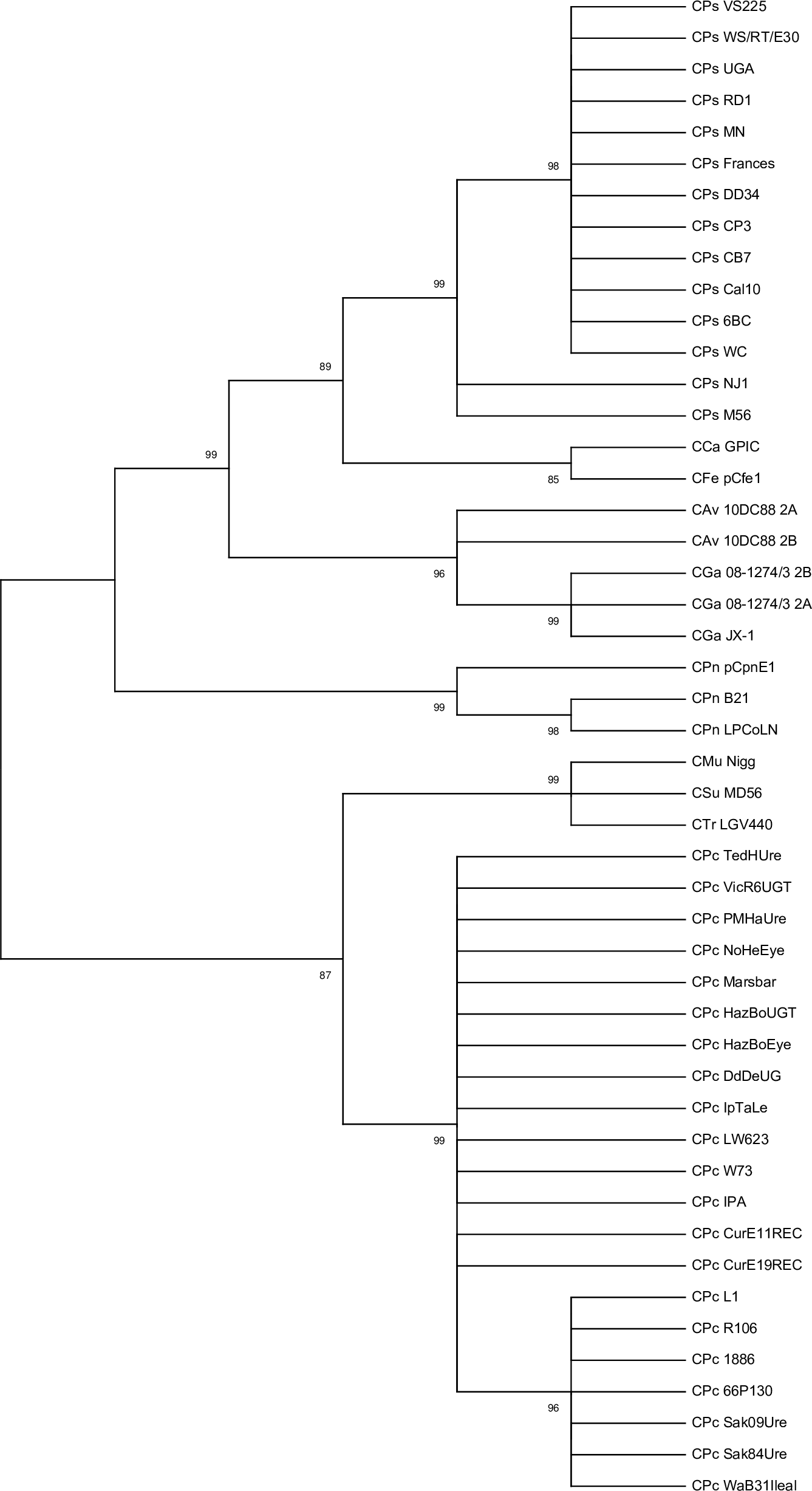

Supplement: S2 Fig — Raw unannotated MEGA X data. (TIF) [file pone.0233298.s002.tif]

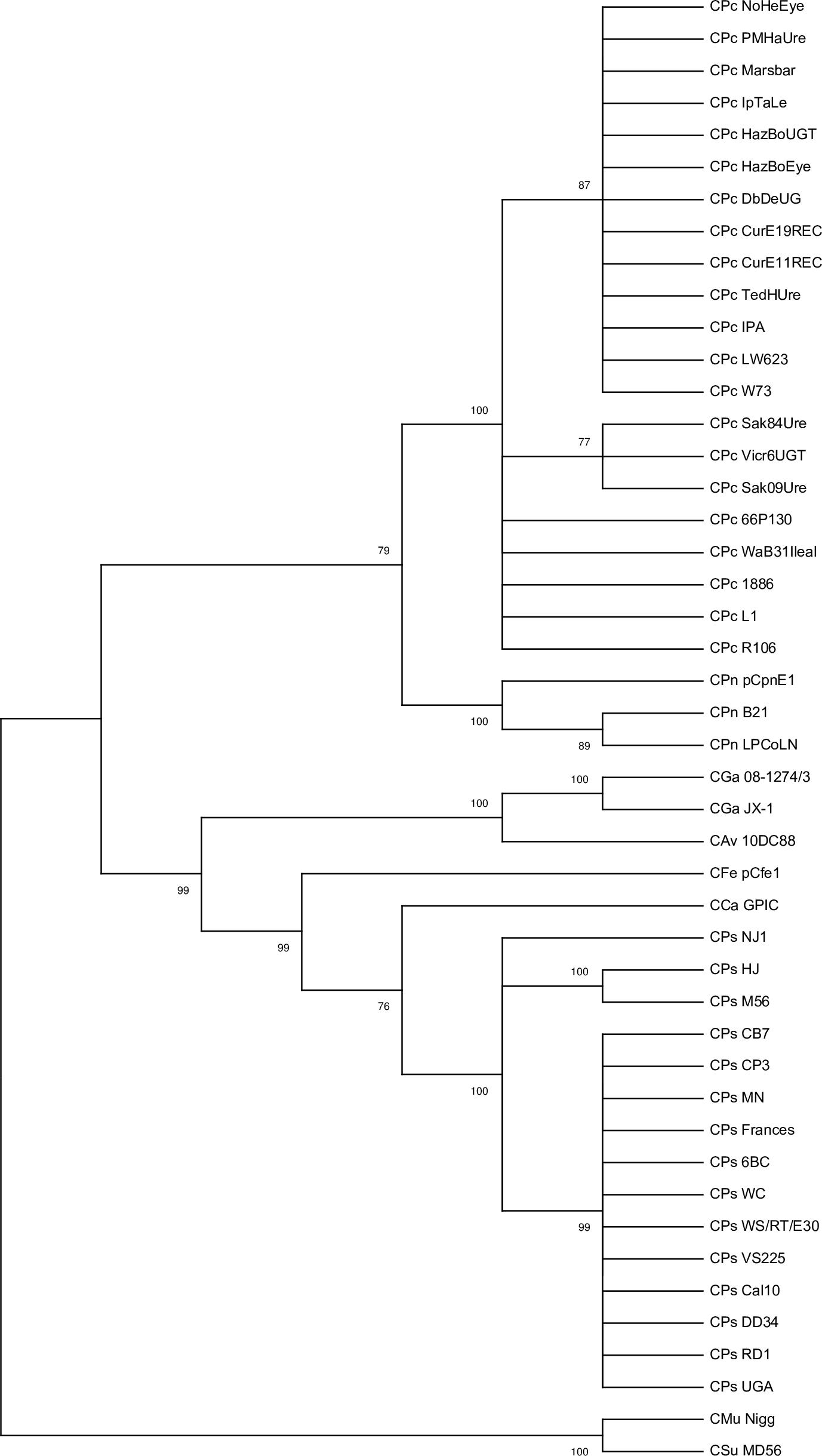

Supplement: S3 Fig — Raw unannotated MEGA X data. (TIF) [file pone.0233298.s003.tif]

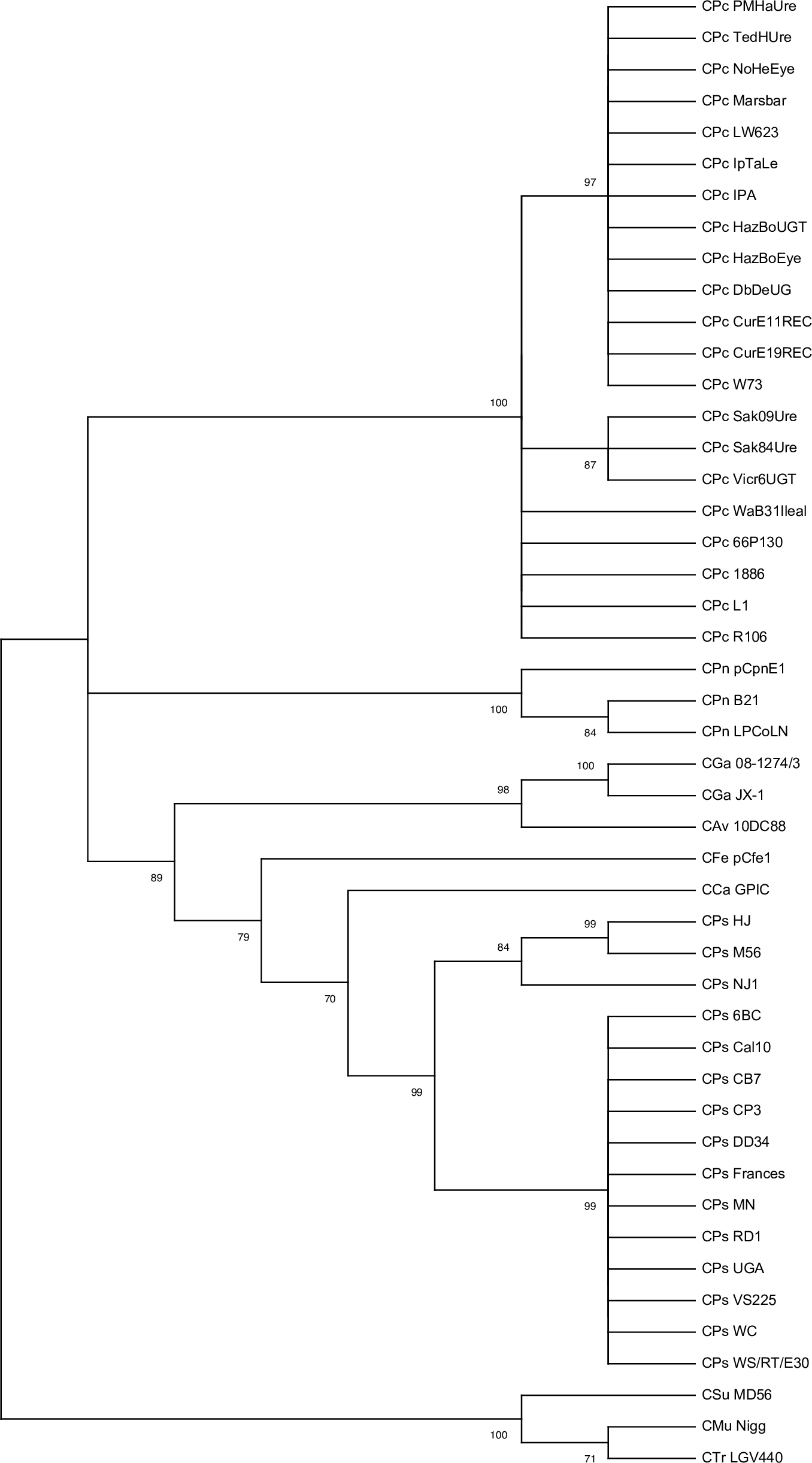

Supplement: S4 Fig — Raw unannotated MEGA X data. (TIF) [file pone.0233298.s004.tif]

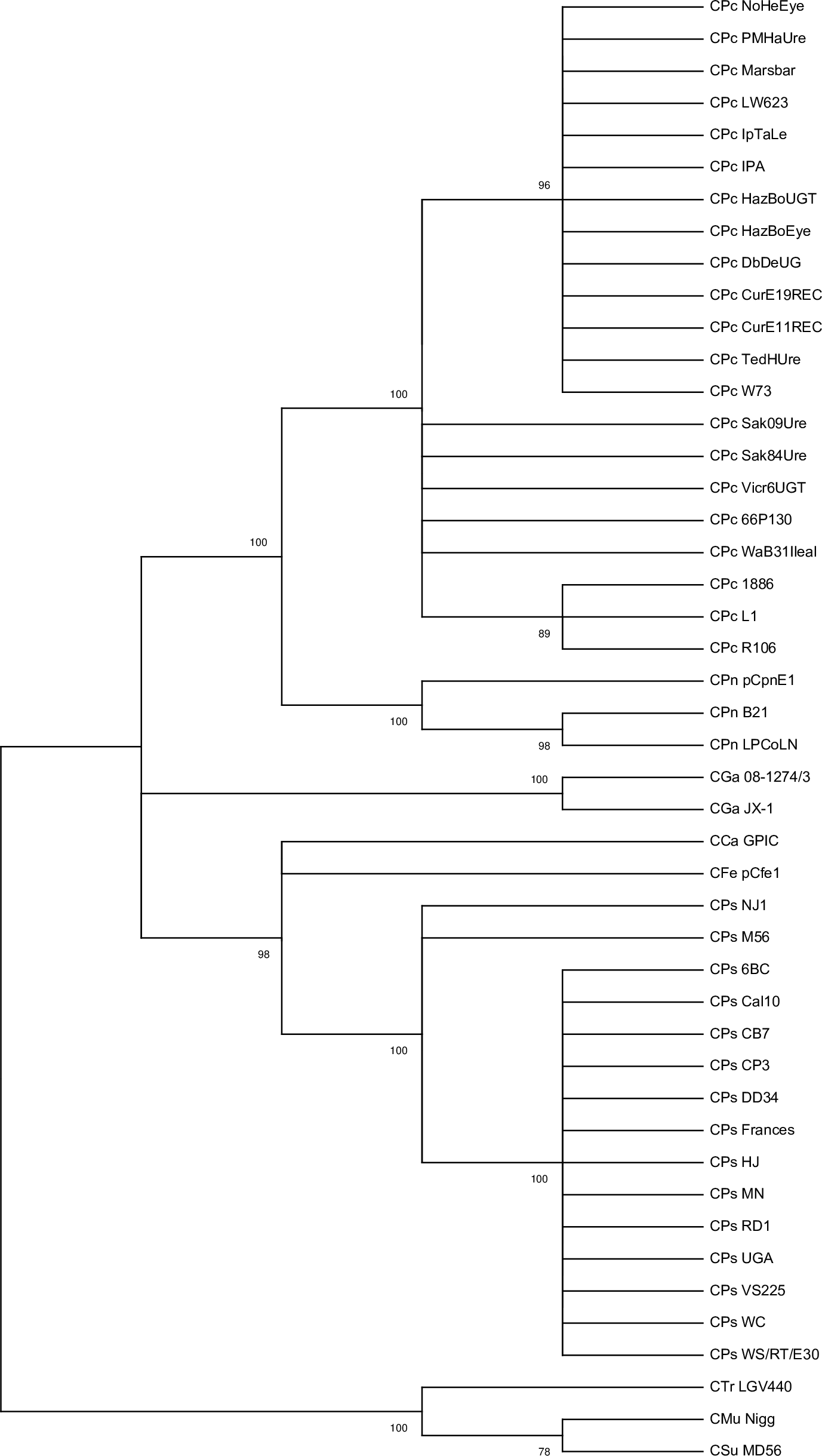

Supplement: S5 Fig — Raw unannotated MEGA X data. (TIF) [file pone.0233298.s005.tif]

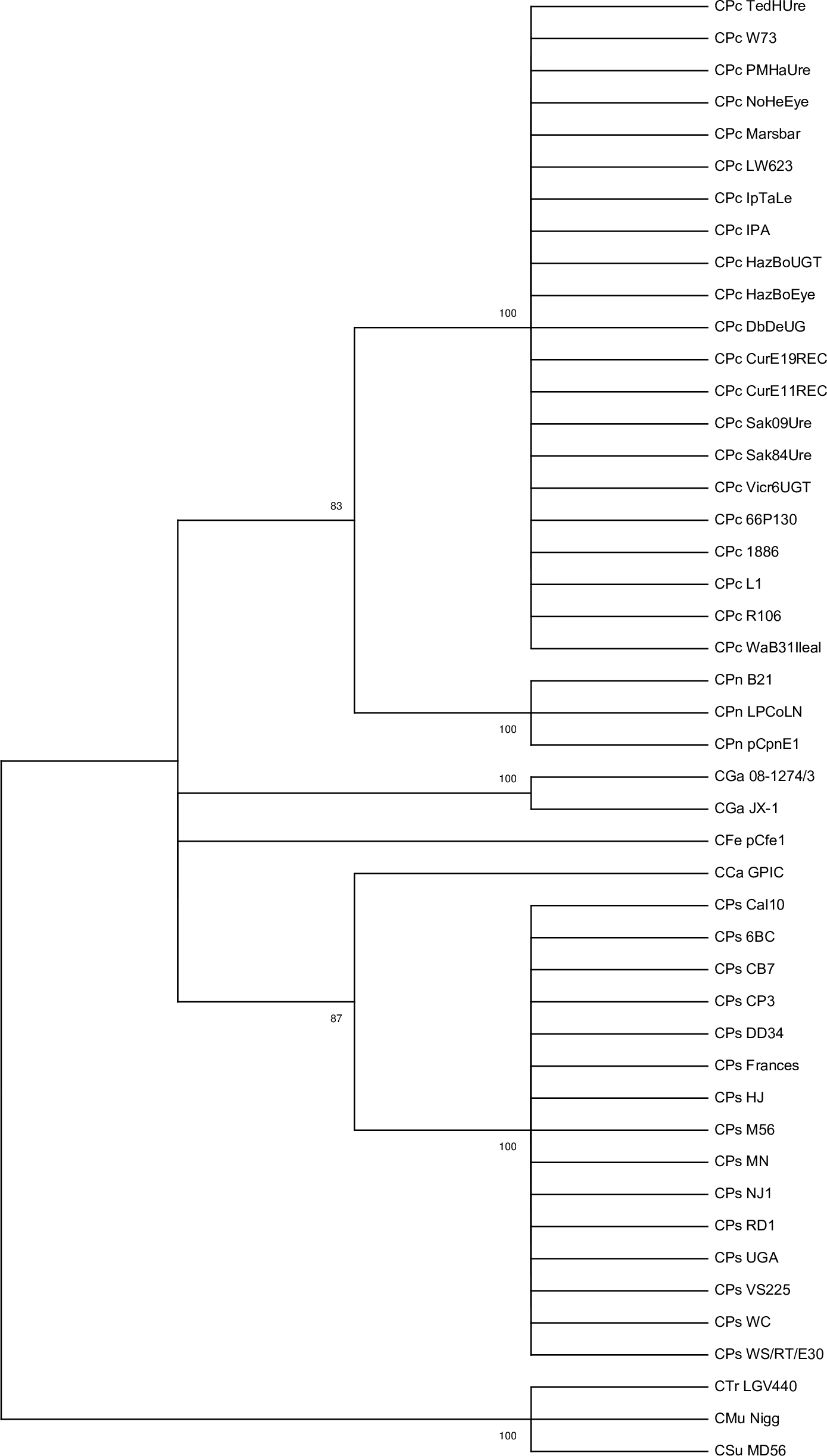

Supplement: S6 Fig — Raw unannotated MEGA X data. (TIF) [file pone.0233298.s006.tif]

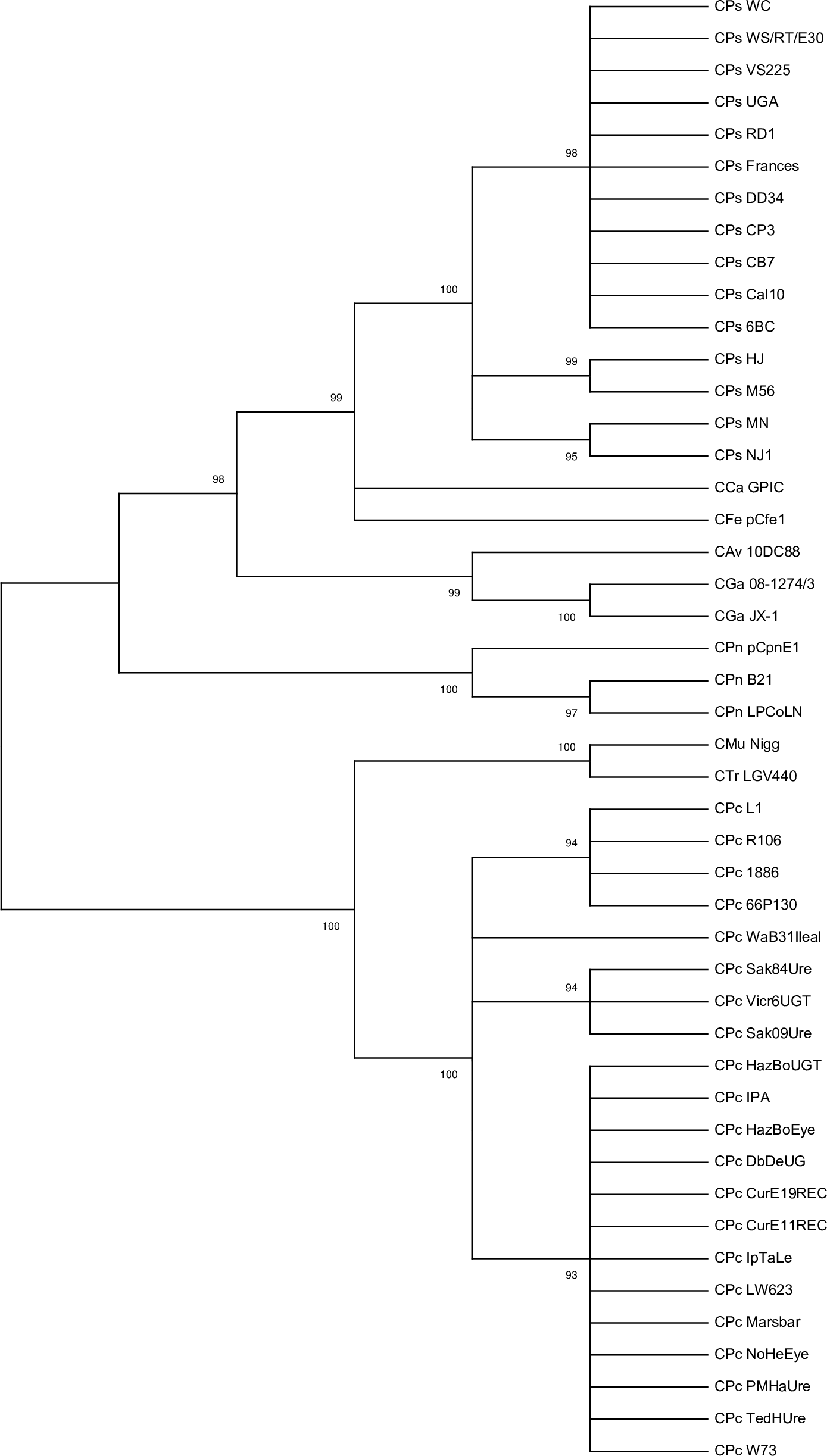

Supplement: S7 Fig — Raw unannotated MEGA X data. (TIF) [file pone.0233298.s007.tif]

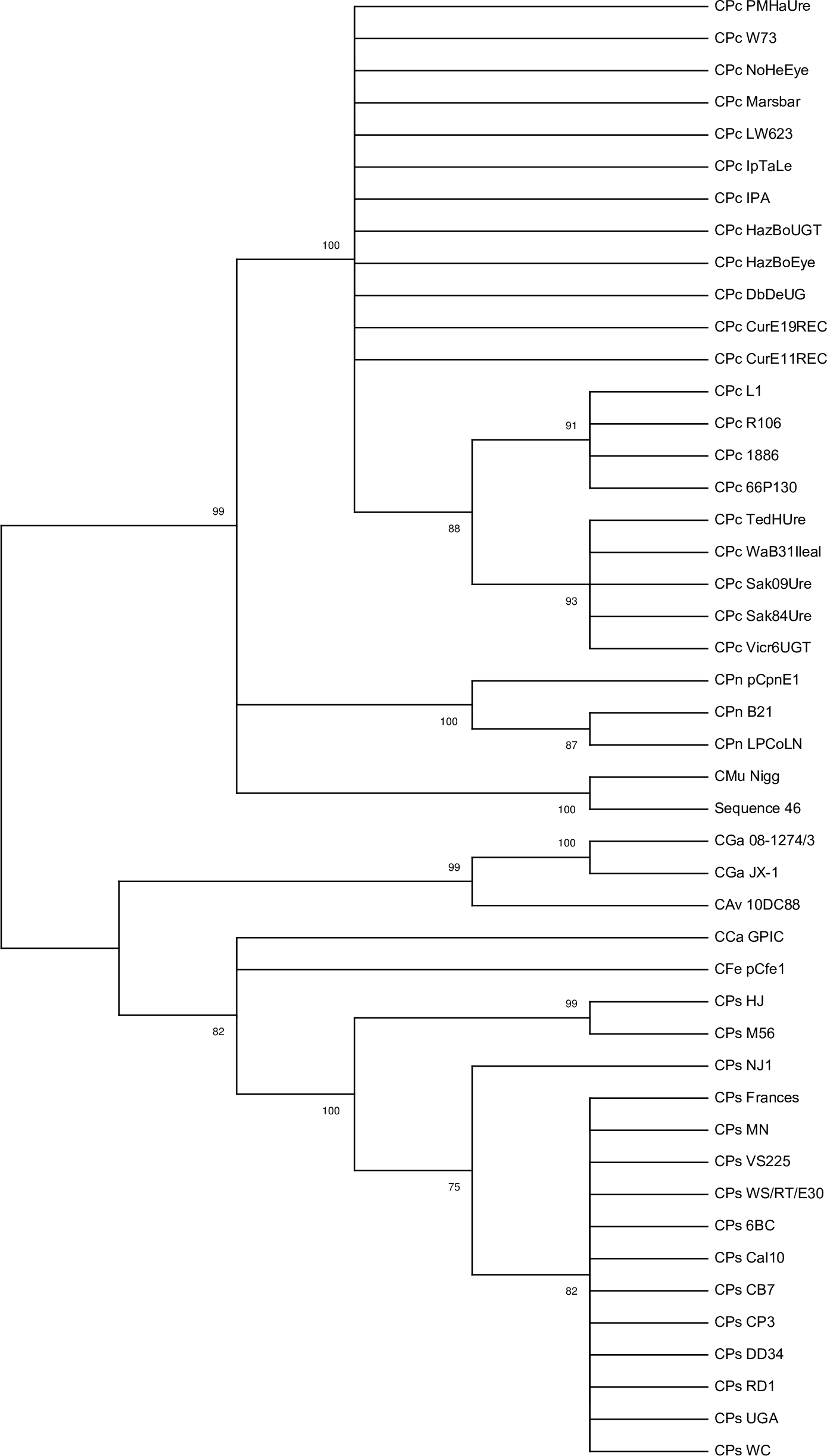

Supplement: S8 Fig — Raw unannotated MEGA X data. (TIF) [file pone.0233298.s008.tif]
